# Medical Comorbidities and Association With Mortality Risk in Alzheimer’s Disease: Population-Based Study of 132,405 Geriatric Inpatients

**DOI:** 10.7759/cureus.8203

**Published:** 2020-05-19

**Authors:** Ting Yu Yen, Nitya Beriwal, Pawandeep Kaur, Virendrasinh Ravat, Rikinkumar S Patel

**Affiliations:** 1 Medicine, Poznan University of Medical Sciences, Poznan, POL; 2 Medicine, Lady Hardinge Medical College, New Delhi, IND; 3 Medicine, Sri Guru Ram Das Institute of Medical Sciences and Research, Amritsar, IND; 4 Internal Medicine, Krishna Institute of Medical Sciences, Karad, IND; 5 Psychiatry, Griffin Memorial Hospital, Norman, USA

**Keywords:** alzheimer’s disease, alzheimer’s dementia, dementia, medical comorbidities, mortality, diastolic heart failure, congestive cardiac failure, metastatic cancer

## Abstract

Objectives

We used the Nationwide Inpatient Sample (NIS) to identify the demographic predictors and study the impact of chronic comorbidities on the risk of in-hospital mortality in Alzheimer’s disease (AD).

Methods

We included 132,405 AD patients from the NIS (2012-2014). We used descriptive statistics to discern the differences in demographics and comorbidities by in-hospital mortality. Logistic regression analysis was used to evaluate the predictors and impact of comorbidities that increase the risk of association with in-hospital mortality.

Results

The in-hospital mortality in AD inpatients is 1.69%, and a greater proportion were female (58.4%) and white (81.5%). Male and hispanic had a higher mortality risk than their counterparts. Hypertension (72%) is the most prevalent comorbidity. Congestive cardiac failure (CCF) and renal failure were significantly associated with a higher risk of in-hospital mortality in AD inpatients by 1.4 and 1.5 times, respectively. Psychiatric comorbidities (depression 20.4%, and psychosis 21.4%) were prevalent in AD inpatients but were negatively associated with mortality. Comorbid tumors without metastasis (1.2%) and metastatic cancer (0.3%) were least prevalent but significantly increased the risk of in-hospital mortality by 1.6 times and 2.2 times, respectively.

Conclusion

CCF and renal failure were significantly associated with a higher risk of in-hospital mortality in AD patients. Less prevalent comorbidities, tumors with/without metastasis increased in-hospital mortality by 59% to 117%. An integrated care model is required to manage comorbidities in AD patients to improve health-related quality of life and reduce morbidity and mortality.

## Introduction

Alzheimer’s disease (AD) is a progressive neurodegenerative disease that occurs due to the accumulation of abnormal protein folding inside the brain. AD accounts for 60% to 80% of the dementia cases [1]. Genetic mutation is mainly involved in early-onset AD patients, and the combination of genetic, environmental, and lifestyle factors are linked to AD [2]. Aging has been known as the most important risk factor, as the number of patients with AD doubles every five years after the age of 65, and above 85 consists of about one-third of the AD population [2]. In 2020, it is estimated that more than 5.5 million Americans suffer from AD and it is expected to increase to 14 million by 2060 [3]. An estimated annual cost per AD patient is $41,000 to $56,000, with a total cost of $159 billion to $215 billion nationwide in 2010 [4].

Among the AD patients, beta-amyloid plaques and the neurofibrillary tangles in the brain are more widespread and predominant than the elderly patients without AD [5]. These changes in the brain impair the normal neuronal functioning and ultimately lead to disconnections with other neurons [2]. AD is the sixth leading cause of death in the United States (US), and the fifth cause for those aged above 65 years [6]. In 2018, there were 122,019 deaths among those suffering from AD, according to the Centers for Disease Control and Prevention (CDC). However, the number of death from dementia was much higher, about 266,957 in total [7].

Bronchopneumonia is the most common cause of death in a patient with AD due to impaired swallowing ability, inability to clear airways secretions, apathies, and decreased immune function may all be crucial as the underlying causes for bronchopneumonia [8]. This is followed by circulatory system disease with ischemic heart disease accounting for more than 60% of deaths and cancer. Lack of oxygen supply in pneumonia patients with AD may contribute to non-symptomatic coronary atherosclerosis resulting in higher inpatient mortality [8]. Several studies have demonstrated an inverse relationship between neurodegenerative disease and cancer; however, underlying pathology behind this remains unknown [9].

We used the US national hospital data to identify the demographic predictors of in-hospital mortality in AD inpatients and the impact of chronic comorbidities on the risk of in-hospital mortality.

## Materials and methods

Data source

Cross-sectional data analysis was done using the Nationwide Inpatient Sample (NIS, 2012 to 2014) [10]. This dataset includes clinical information during the hospitalization from 4,400 non-federal hospitals and covers about 44 states in the US [10]. Diagnostic information is coded using the International Classification of Diseases, ninth edition (ICD-9) diagnostic codes, and Clinical Classification Software (CCS) codes. The NIS is a publicly available de-identified database and so doesn’t require approval from the institutional review board [10].

Inclusion criteria and outcome variables

We included 132,405 inpatients with a primary discharge diagnosis of AD using ICD-9 code 331.0. Demographic variables included were age, sex, and race (white, black, hispanic, and others) [11]. The chronic comorbidities including diabetes, hypertension, congestive cardiac failure (CCF), chronic pulmonary disease, renal failure, depression, psychosis, and tumor with and without metastasis were identified using ICD-9 or CCS diagnosis codes [11]. The in-hospital mortality in the study population is all-cause [11].

Statistical analysis

We used descriptive statistics to discern the differences in demographics and comorbidities by in-hospital mortality. Logistic regression analysis was used to evaluate the demographic predictors and impact of comorbidities that increases the risk of association with in-hospital mortality. A P-value of less than 0.01 was considered statistical significance in our data analysis models that were done in the Statistical Package for the Social Sciences (SPSS), version 26 (IBM Corporation, Armonk, NY).

## Results

**Figure 1:**
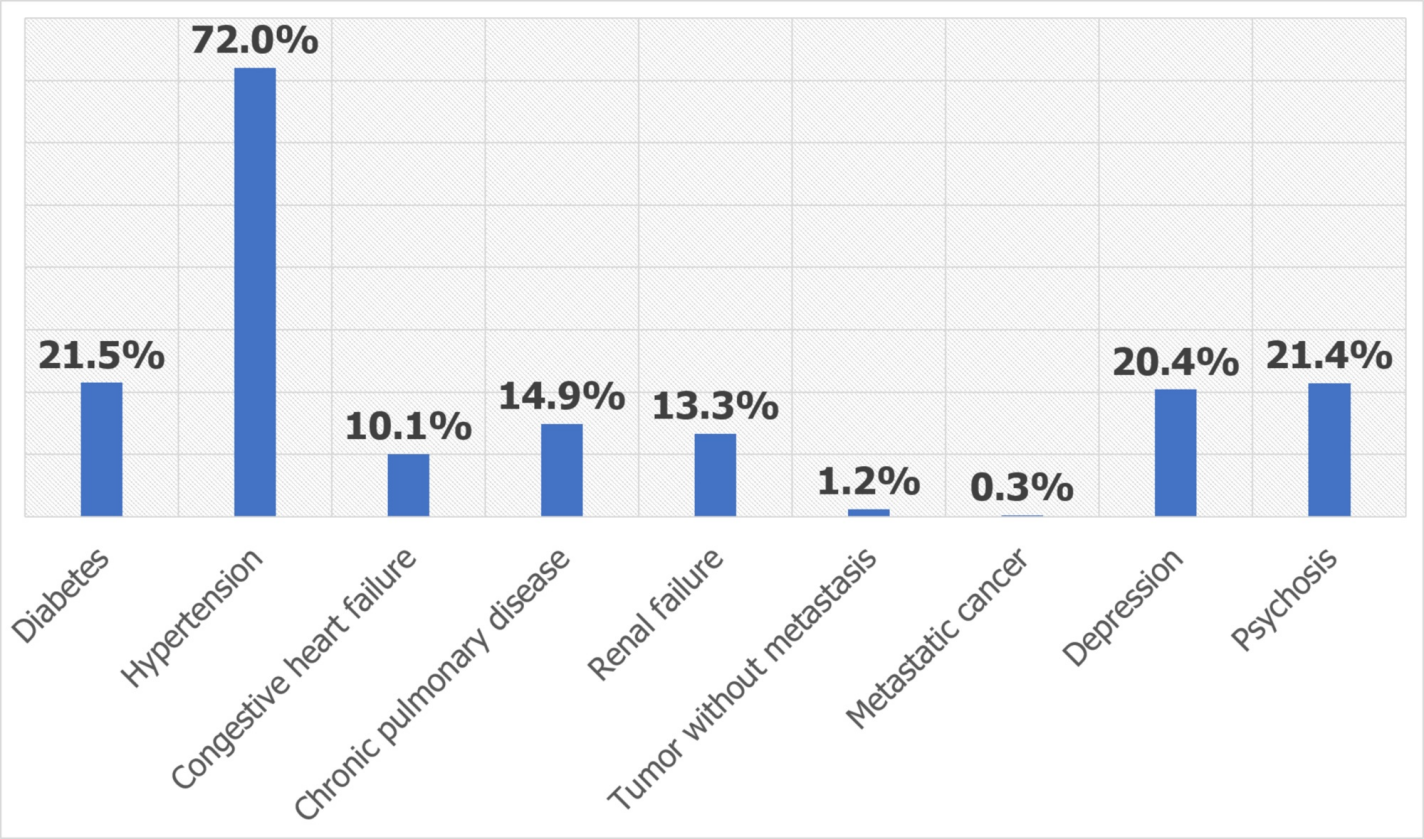
Prevalence of comorbidities in Alzheimer’s disease inpatients

We analyzed a total sample of 132,405 AD inpatients with an in-hospital mortality of 1.69% (N = 2,245). The majority of the AD inpatients were female (58.4%) and white (81.5%). The most prevalent chronic comorbidities were hypertension (72%), as shown in Figure 1.

Risk factors for in-hospital mortality

The mean age of AD inpatients who died during hospitalization were older (83.5 vs. 80.6 years). Males had 1.2 times higher odds for deaths (95% CI 1.069-1.276) than females. Though 78.2% of Whites died during hospitalization, in the regression model, hispanics had two times (95% CI 1.617-2.210) higher risk of mortality compared to whites.

CCF and renal failure were significantly associated with a higher risk of in-hospital mortality in AD inpatients by 1.4 to 1.5 times. Psychiatric comorbidities (depression 20.4%, and psychosis 21.4%) were prevalent in AD inpatients but were negatively associated with mortality. Comorbid tumor without metastasis (1.2%) and metastatic cancer (0.3%) were least prevalent but significantly increases the risk of in-hospital mortality by 1.6 times (95% CI 1.193-2.128) and 2.2 times (95% CI 1.285-3.653), respectively, after controlling for demographic and other comorbidities as shown in Table 1.

**Table 1:** Risk factors of in-hospital mortality in Alzheimer’s disease inpatients N: number of patients; OR: odds ratio

Variable	In-hospital mortality, %		Logistic regression model		
	No	Yes	OR	95% CI	P-value
Total (N)	130,155	2,245	-	-	-
Mean age, years (SD)	80.6 (7.72)	83.5 (6.19)	1.06	1.055–1.070	<0.001
Sex					
Male	41.6	45.9	1.17	1.069–1.276	<0.001
Female	58.4	54.1	Reference		
Race					
White	81.5	78.2	Reference		
Black	9.7	7.7	0.96	0.811–1.130	0.609
Hispanic	5.1	8.9	1.89	1.617–2.210	<0.001
Other	3.8	5.3	1.49	1.228–1.822	<0.001
Comorbidities					
No comorbidities	-	-	Reference		
Diabetes	21.5	19.8	1.13	1.013–1.260	0.029
Hypertension	72.3	53.0	0.39	0.356–0.427	<0.001
Congestive cardiac failure	10.0	13.6	1.41	1.237–1.599	<0.001
Chronic pulmonary disease	15.0	11.8	0.84	0.733–0.956	0.009
Renal failure	13.3	17.6	1.48	1.310–1.661	<0.001
Tumor without metastasis	1.2	2.2	1.59	1.193–2.128	0.002
Metastatic cancer	0.3	0.7	2.17	1.285–3.653	0.004
Depression	20.5	14.7	0.68	0.595–0.766	<0.001
Psychosis	21.6	10.0	0.47	0.404–0.539	<0.001

## Discussion

In our inpatient study, the mean age of AD inpatients who died during hospitalization was 83.5 years. As per a study, AD is prevalent in the age above 90 years and is projected to increase rapidly [12]. Male AD patients had a 17% higher risk of mortality, which is supported by the Framingham heart study; however, the causal relationship remains speculative [13]. Our AD inpatients from the hispanic race group had higher in-hospital mortality increased by 89%. As per the National Alzheimer’s Coordinating Center data from 30 AD centers in the US (N = 30,916) found that Latino and blacks with AD had a longer survival time compared to the whites [14]. Yet, this study had some limitations, including a lack of uniform process for data acquisition and selection bias [14]. Whites have better access to healthcare for the management of comorbidities and have a higher rate of nursing home placement due to which in our study we found that whites had a lower risk of mortality compared to minority groups [15].

Hypertension is the most prevalent chronic comorbidities (72%), followed by diabetes (21.5%), psychosis (21.4%), and depression in our study AD inpatients. In a study comparing the comorbidities of young-onset versus late-onset AD, circulatory disease and hypertension were most prevalent in both [16]. This is followed by psychiatric disorders, and endocrine and metabolic disorders, and diabetes [16]. Notably, the late-onset AD patients had a significantly higher prevalence rate of hypertension (61.2% vs. 35% in early-onset) and diabetes (20.9% vs. 7.8% in early-onset) [16]. Aging increases the prevalence rate of these chronic comorbidities, which are also risk factors for AD’s progression [17]. Lung diseases, other forms of heart disease, and disease of the genitourinary system are remarkable comorbidity in AD patients; however, they are less prevalent as also seen in our study [16].

The macrovascular and microvascular damage from hypertension and diabetes, consequently, lead to CCF and renal failure. Glomerular hyperfiltration, overactive renin-angiotensin-aldosterone system, and renal dynamic changes may all contribute to renal disease [18]. In our study, we did not find a significant association between hypertension and diabetes and in-hospital mortality, but renal failure and CCF significantly increases mortality risk by 48% and 41%, respectively. Some studies in Parkinson’s disease found that depression and psychosis worsen hospitalization outcomes and disease severity, but this was not seen in our AD patients and was not associated with increased mortality risk [19,20].

Multiple studies suggested that AD is inversely associated with cancer-specific mortality [9,21]. The paired-helical-filament (PHF)-tau tangles were lower in AD patients with cancer, and PHF-tau tangles correlate to cognitive impairment and dementia severity [21]. Cancer patients may have enhanced immune function to reliant AD progression, better lifestyle choices, and possible PHF-tau protein reduction from chemotherapy [21]. However, our study has demonstrated that metastatic and non-metastatic tumor increases the risk of in-hospital mortality by 117% and 59%, respectively, after controlling for demographic confounders and chronic comorbidities.

There are some limitations to our study. Our inpatient population was predominantly white and so may lack population diversity and may not represent the general population. Second, the healthcare availability to minority populations may be underreported in our study sample. Lastly, the inconsistency and reporting bias about using the ICD-9 codes in the database, and lack of causal relationship between mortality in AD and comorbidities. Regardless of this, we have used a large sample population from the hospitals across 44 states in the US with national representation and found an epidemiological association between chronic comorbidities worsening mortality risk in AD patients.

## Conclusions

About four-fifth of AD inpatients were white and prevalent comorbidities included hypertension and diabetes. CCF and renal failure were significantly associated with a higher risk of in-hospital mortality in AD patients by 41% and 48%, respectively. In addition, the less prevalent comorbid tumor with/without metastasis also increased the in-hospital mortality by 59% to 117%. Psychiatric comorbidities were prevalent (20% to 21%) but were negatively associated with mortality risk. An integrated care model is required to manage medical and psychiatric comorbidities in AD patients to improve health-related quality of life and reduce morbidity and mortality.

## References

[1] (2020). Alzheimer's and dementia: facts and figures. https://www.alz.org/alzheimers-dementia/facts-figures.

[2] (2020). What causes alzheimer's disease?. https://www.nia.nih.gov/health/what-causes-alzheimers-disease.

[3] (2020). Alzheimer's disease fact sheet. https://www.nia.nih.gov/health/alzheimers-disease-fact-sheet.

[4] Hurd MD, Martorell P, Delavande A, Mullen KJ, Langa KM (2013). Monetary costs of dementia in the United States. N Engl J Med.

[5] (2020). Causes of dementia. https://www.dementia.org.au/about-dementia/dementia-research/causes-of-dementia.

[6] (2020). Leading causes of death and numbers of deaths, by age: United States, 1980 and 2017. https://www.cdc.gov/nchs/data/hus/2018/007.pdf.

[7] (2020). National vital statistics reports. https://www.cdc.gov/nchs/data/nvsr/nvsr68/nvsr68_02-508.pdf.

[8] Heun R, Schoepf D, Potluri R, Natalwala A (2013). Alzheimer's disease and co-morbidity: increased prevalence and possible risk factors of excess mortality in a naturalistic 7-year follow-up. Eur Psychiatry.

[9] Romero JP, Benito-Leon J, Louis ED, Bermejo-Pareja F (2014). Alzheimer's disease is associated with decreased risk of cancer-specific mortality: a prospective study (NEDICES). J Alzheimers Dis.

[10] (2020). Overview of the national (nationwide) inpatient sample. https://www.hcup-us.ahrq.gov/nisoverview.jsp.

[11] (2020). NIS description of data elements. https://www.hcup-us.ahrq.gov/db/nation/nis/nisdde.jsp.

[12] Kato K, Shirosita K, Kurosawa S (1990). Staphylococcal enterotoxin a induced interferon (ifn)-gamma production in spleen cells from bcg-immunized mice: the ifn production is dependent on leukotriene c4 but not dependent on interleukin 2. Immunobiology.

[13] Chene G, Beiser A, Au R, Preis SR, Wolf PA, Dufouil C, Seshadri S (2015). Gender and incidence of dementia in the Framingham Heart Study from mid-adult life. Alzheimer's Dement.

[14] Mehta KM, Yaffe K, Perez-Stable EJ, Stewart A, Barnes D, Kurland BF, Miller BL (2008). Race/ethnic differences in ad survival in US Alzheimer's disease centers. Neurology.

[15] Stern Y, Tang MX, Albert MS (1997). Predicting time to nursing home care and death in individuals with Alzheimer disease. JAMA.

[16] Gerritsen AA, Bakker C, Verhey FR, de Vugt ME, Melis RJ, Koopmans RT, 4C study team (2016). Prevalence of comorbidity in patients with young-onset alzheimer disease compared with late-onset: a comparative cohort study. J Am Med Dir Assoc.

[17] Norton S, Matthews FE, Barnes DE, Yaffe K, Brayne C (2014). Potential for primary prevention of alzheimer's disease: an analysis of population-based data. Lancet Neurol.

[18] Lin YC, Chang YH, Yang SY, Wu KD, Chu TS (2018). Update of pathophysiology and management of diabetic kidney disease. J Formos Med Assoc.

[19] Imran S, Patel RS, Onyeaka HK (2019). Comorbid depression and psychosis in Parkinson's disease: a report of 62,783 hospitalizations in the United States. Cureus.

[20] Patel RS, Makani R, Mansuri Z, Patel U, Desai R, Chopra A (2017). Impact of depression on hospitalization and related outcomes for Parkinson's disease patients: a nationwide inpatient sample-based retrospective study. Cureus.

[21] Yarchoan M, James BD, Shah RC (2017). Association of cancer history with Alzheimer's disease dementia and neuropathology. J Alzheimers Dis.

